# Iron-On Wearable Electronics through Liquid Metal Adhesive Composites

**DOI:** 10.1021/acsami.5c13752

**Published:** 2025-11-09

**Authors:** John Joyce, Brittan T. Wilcox, Anna Ingram, Michael D. Bartlett

**Affiliations:** † Mechanical Engineering, Soft Materials and Structures Lab, 1757 Virginia Tech, Blacksburg, Virginia 24061, United States; ‡ Macromolecules Innovation Institute, 1757 Virginia Tech, Blacksburg, Virginia 24061, United States

**Keywords:** liquid metal composites, stretchable conductors, wearable electronics, e-textiles, soft circuit integration, fabric adhesion, thermoplastic polyurethane (TPU)

## Abstract

E-textiles and wearable electronics can enable diverse applications from health care and environmental monitoring to robotics and human-machine interfaces. These technologies demand circuity that is flexible and stretchable while being able to integrate with functional components and deformable substrates like fabrics. Therefore, stretchable conductors and processing techniques that tightly integrate these diverse components both electronically and mechanically are important for these emerging devices. Here, we create composites of liquid metal (LM) microdroplets within a thermoplastic polyurethane (TPU) matrix that is stretchable (greater than 600% strain at break), adhesive to various common fabrics via heat transfer (toughness up to 6400 J m^–2^), and electrically conductive as prepared (up to 8.0 × 10^5^ S m^–1^). By using a thermoplastic matrix, the LM-TPU composites can be reprocessed, making them applicable to hot melt adhesion while the LM droplets enable electrical conductivity. The LM-TPU composites create soft conductors that electrically and mechanically integrate via heat transfer with rigid components and fabrics to create functional soft circuits. This enables flexible, electrically conductive composites that can be readily integrated for applications in wearable circuits and e-textiles.

## Graphical abstract

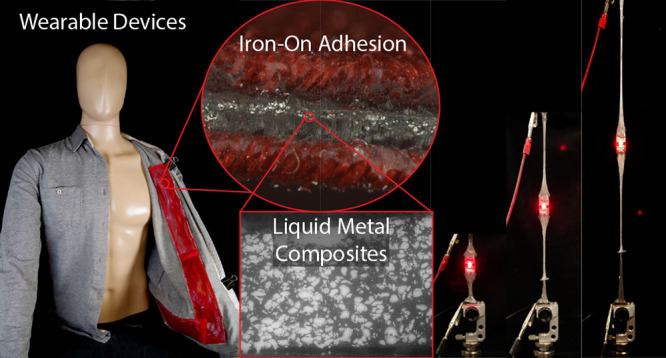

## Introduction

Wearable electronics are becoming incorporated into everyday life through devices like watches or wrist straps,^1–3^ hats,^4^ and glasses.^5^ While commercial wearable devices have made advances to be more conformal, soft electronics, which are inherently stretchable and adaptable, can enable a step change toward more conformal wearable technology. To make more conformal and robust wearable electronics, one focus has turned toward integrating soft circuit materials into fabrics and textiles through the use of elastomers and films which can maintain conformal contact with curvilinear surfaces.^6–12^ Several approaches for processing these circuits have been explored including spray coating,^13–18^ electrode deposition,^19^ creating conductive smart fibers that can be woven into a fabric,^20–27^ and adhering electronics to a fabric^28–31^ and to skin.^32–34^ In these strategies, a circuit must be flexible and elastic so that it remains functional while undergoing deformation during use.

One approach to improve the flexibility and elasticity of wearable electronics is through the use of liquid metal (LM) and LM composites.^35–40^ Eutectic gallium–indium (EGaIn) is a liquid metal at room temperature, allowing it to be incorporated into soft composites that can bend, fold, and stretch while remaining electrically conductive.^41–43^ LM can be incorporated in polymer matrix composites^44–48^ where the pretreatment and size of the LM droplets can change the mechanical and electrical properties of the composite.^49–53^ Additionally, LM can be combined with other inclusions or phases such as metallic particles, metal oxides, or carbon based fillers to create biphasic composites.^54^ These compositions are typically paste-like or viscous mixtures which improves interfacing beyond liquid metal alone, but are not typically inherently adhesive and can be smeared, highlighting needs for enhanced integration schemes.^40,55^ Many LM composites also need to be “sintered” to become electrically activated through techniques like “mechanical activation” by straining the material to connect the dispersed droplets within the polymer matrix.^6,56–59^ Alternatively, enabling LM composites with inherent electrical conductivity and adhesion through material composition and processing strategies can streamline fabrication and integration, yielding robust soft electronic systems.

Different polymer matrices also provide varying properties and processing techniques for LM composites. Chemically cross-linked thermoset matrices like silicone elastomers utilize strong covalent bonds for cross-links between the molecules to create robust LM composites,^60,61^ however they can not be reprocessed. Thermoplastic elastomers like block copolymers of styrene-isoprene-styrene (SIS) and thermoplastic polyurethanes (TPU), which are physically cross-linked networks, can also be made into composites through the incorporation of LM inclusions,^62–67^ and due to their physically cross-linked network can be reprocessed. The ability to reprocess thermoplastic elastomers opens the possibility for versatile manufacturing techniques which can enable strong integration with different substrates. This includes methods such as melt reprocessing, which is not possible with thermoset matrices such as silicone elastomers.

Thermoplastic elastomer LM composites have been shown to provide unique characteristics. For example, wet spinning processes with EGaIn can create TPU fibers that are activated through mechanical sintering (up to 1.2 × 10^5^ S m^–1^) which can be woven into fabrics to create soft electronics.^23^ Additionally, LM can be injected directly into hollow TPU fibers to create conductive elastic fibers with shape memory aspects using the thermal deformation of the TPU composite.^20^ Furthermore, LM circuits can be integrated into fabrics by depositing LM through spray-coating onto SIS thermoplastic elastomer sheets^68^ or by casting LM-SIS adhesive composites^69^ and printing biphasic-SIS composites without heat pressing.^70^ A related strategy encapsulated conductive silver inks with thermoplastic elastomer sheets, which were then ironed onto fabrics to provide adhesion.^31^ While these strategies highlight promising pathways for combining conductors with textiles, opportunities remain to develop approaches that simultaneously provide strong fabric integration, stretchability, and multifunctionality in reprocessable systems.

Here, we demonstrate a LM-TPU composite film which is electrically conductive as fabricated with strong, robust adhesion to fabrics via heat transfer. We use an emulsion process to create a viscous mixture of LM microdroplets in a TPU solution which can be cast into a mold, and upon solvent evaporation, forms an elastic sheet (Figure 1a). These composite sheets form soft, stretchable circuitry that can then be reprocessed for integration through heat-pressing or ironing into a wide range of fabrics, where the thermoplastic polymer flows into the fabric weave to create a strong, durable bond (Figure 1b). Furthermore, we can incorporate additional layers of pristine TPU through heat pressing for robust encapsulation and solvent weld in rigid electrical components for functional circuitry. These circuits maintain electrical connections to rigid electronic components while being folded, twisted, and stretched (Figure 1c), enabling their use in iron-on wearable circuits and soft electronics.

**Figure 1:**
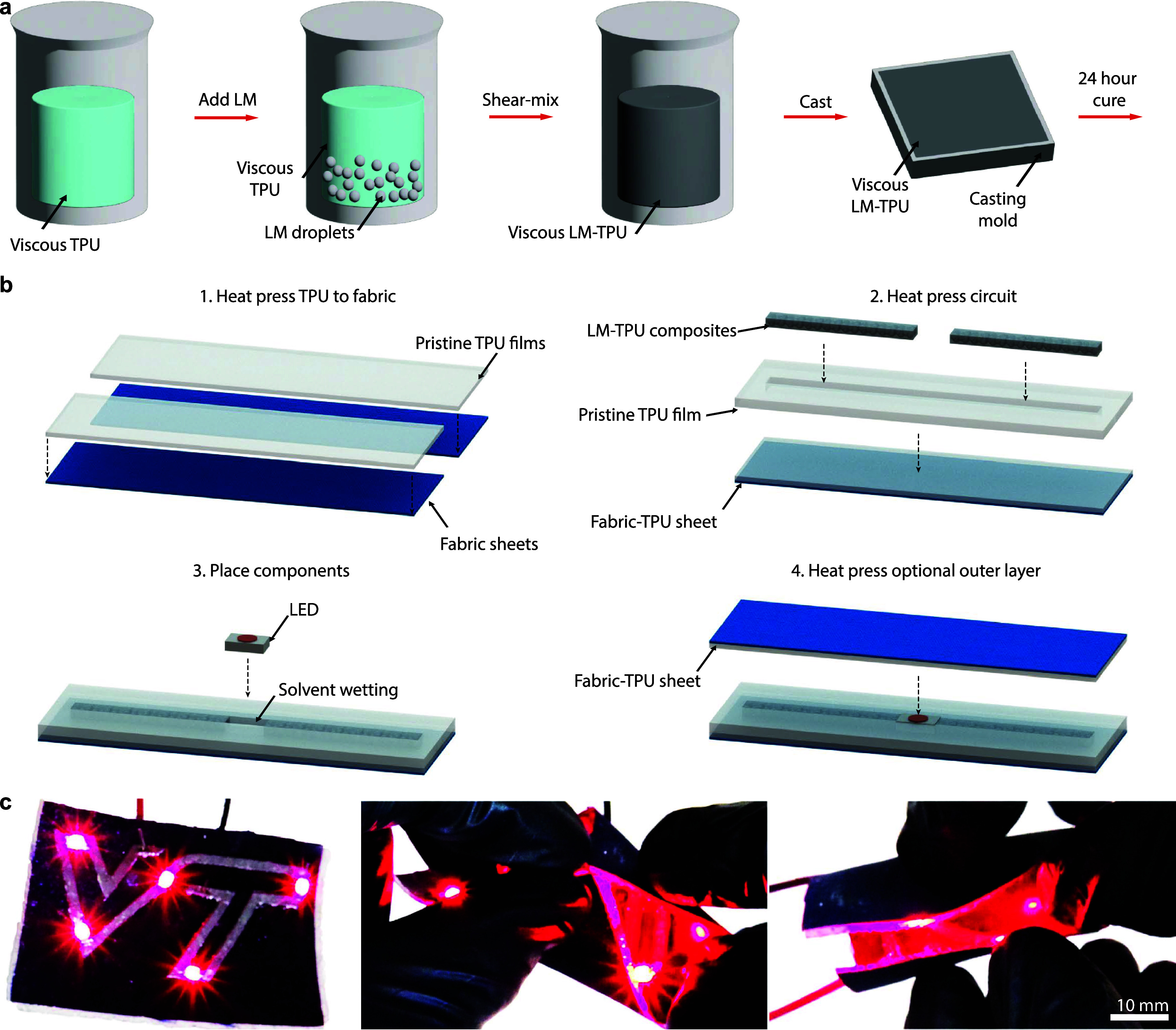
LM-TPU composite fabrication and function. (a) Schematic of LM-TPU composite fabrication process. (b) Processing schematic for integrating LM-TPU composite circuits into fabrics. (c) Images of a LM-TPU composite circuit with LEDs in varying configurations.

## Results and Discussion

### Materials and Processing

The fabrication process begins by creating a soft LM-TPU composite film (Figure 1a). TPU is selected as the polymer matrix as it is both extensible, enabling soft circuits, and has also been employed commercially as a fabric adhesive. First, shear mixing is used to create a viscous TPU solution by dissolving TPU pellets in the solvent tetrahydrofuran (THF). LM is then added to the solution, and a shear mixing process is used to control microdroplet sizing, which affects the overall conductivity of the composite. The volume fraction of LM is defined as ϕ and is calculated for the final film composition without solvent. The LM-TPU mixture is then cast into an open mold of a specified thickness, and the solvent is evaporated for 24 h so that a LM-TPU soft conductive composite film forms. During the evaporation process, the sample is flipped 8 h after initial casting. Flipping the sample provides a more homogeneous dispersion of the LM droplets, preventing gravitational settling of the droplets due to the initially low viscosity of the TPU-THF solution. Details of how the parameters of this process impact electrical conductivity are described below. Pristine TPU is fabricated through the same process, without the incorporation of LM.

### Electrical Conductivity

Electrical conductivity was examined as a function of several material and processing parameters. This includes the volume fraction of LM (ϕ), the droplet size of LM, the thickness of the LM-TPU film, and the process control of LM droplet distribution and settling during formation of the LM-TPU film. Further, the electrical conductivity was measured before and after heat pressing the LM-TPU films into fabrics. A four-point testing setup (Figure 2a) was used to determine the electrical conductivity of all the composites. Electrical conductivity was measured for LM-TPU composites at ϕ = 30, 35, 40, 45, and 60% volume fractions (Figure S1). We find that the percolation of LM droplets begins around ϕ = 35% and increases with higher LM content. For this study we will focus on composites with ϕ = 30, 45, and 60%, which represents a wide range of LM loadings to determine how composition influences composite properties. ϕ = 30% composites are not conductive, as the low volume fraction of EGaIn within the TPU matrix is not adequate to form a conductive network, and thus the LM microdroplets remain discrete. For ϕ = 45 and 60%, electrical conductivities varied based on the three parameters mentioned previously.

**Figure 2:**
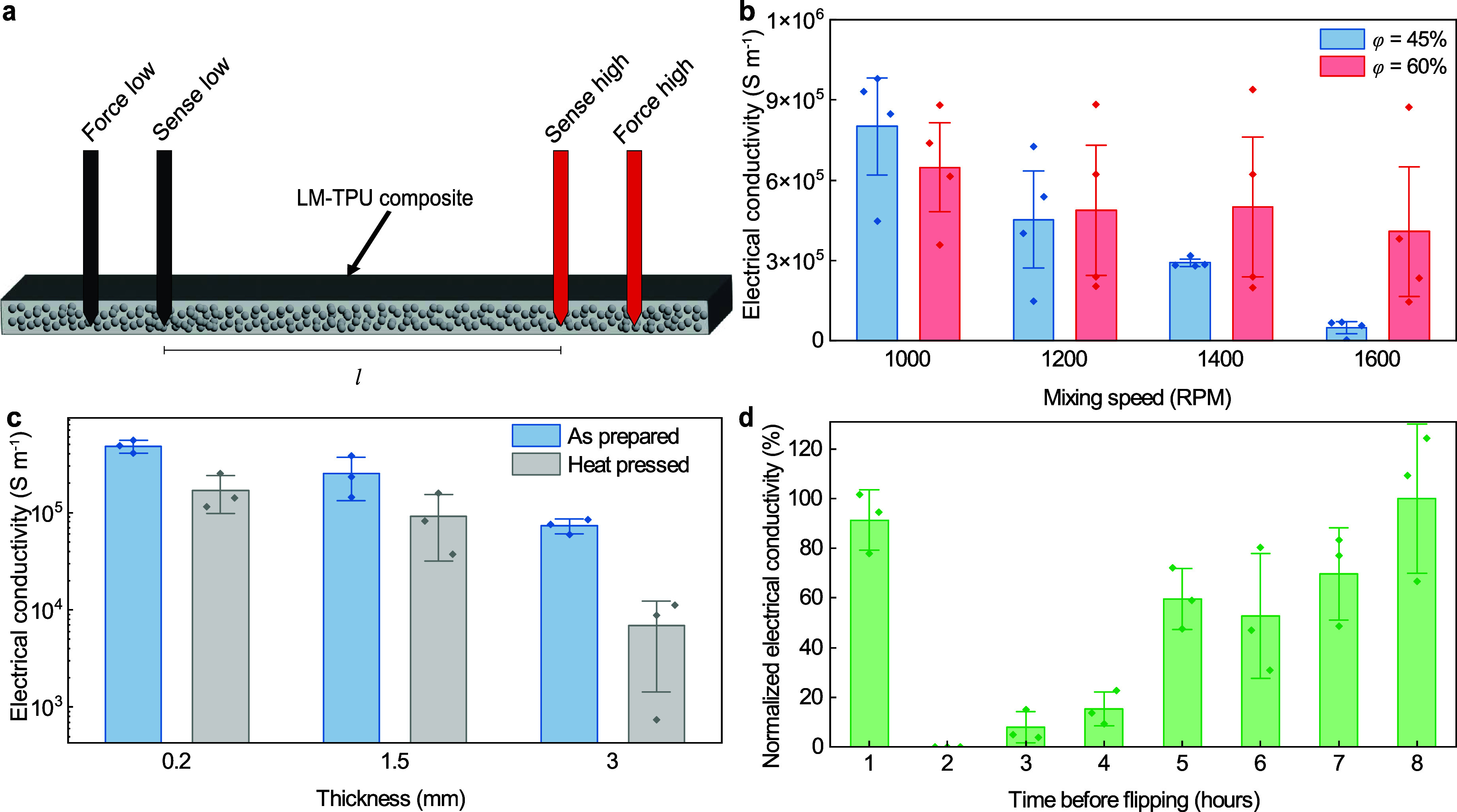
Electrical conductivity of LM-TPU composites. (a) Schematic showing the four-point testing setup. (b) Electrical conductivity for 1.5 mm thick LM-TPU composites with ϕ = 45 and 60% for varying shear mixing speeds. (c) Electrical conductivity for 200 μm, 1.5 mm, and 3 mm thick LM-TPU composites with ϕ = 60%, both as prepared and after heat press processing. (d) Normalized electrical conductivity versus the time before flipping the film during processing (normalized by the 8 h duration) for 1.5 mm thick LM-TPU composites with ϕ = 60%. Data represent the mean ± 1 s.d. (*n* = 3).

EGaIn droplet size was controlled within the matrix by controlling the shear mixing conditions during emulsion processing, where the rotational speed of the planetary centrifugal mixer is set to 1000, 1200, 1400, or 1600 rpm. Smaller droplet sizes are obtained through higher mixing speeds, with a droplet major diameter as low as 76.0 ± 20.8 μm for higher mixing speeds of 1600 rpm at ϕ = 60% compared to a droplet major diameter of 115.8 ± 31.8 μm alongside larger agglomerations of settled bulk LM for lower mixing speeds of 1000 rpm (Figure S2). SEM cross-section images of ϕ = 60% LM-TPU composites also reveal that greater homogeneity is achieved through mixing at 1600 rpm compared to 1000 rpm for our composites (Figure S2). Using different mixing speeds to change droplet size also yields varying electrical conductivities (Figure 2b). ϕ = 45% composites have a conductivity of up to 8.0 × 10^5^ S m^–1^ at low mixing speeds, but this decreases (as low as 4.84 × 10^4^ S m^–1^) as the mixing speed is increased. ϕ = 60% tended to have similar conductivity compared to the ϕ = 45% composites at lower mixing speeds but a higher conductivity (4.65 × 10^5^ S m^–1^) when mixed at higher mixing speeds. Due to these results, 1600 rpm will be used in this work to aid in achieving a uniform dispersion of LM droplets in the composite.

Electrical conductivity was also tested for LM-TPU films with thicknesses of 0.2, 1.5, and 3 mm for ϕ = 60% composites (Figure 2c). An as-prepared conductivity measurement was taken on the surface of the composite after solvent evaporation in the mold, and a second measurement was made after the composite had been adhered to fabric via heat transfer. We find that electrical conductivity is lower for LM-TPU films with greater thickness. Additionally, across all samples heat pressing does tend to lower electrical conductivity slightly. We observe that for 0.2 and 1.5 mm thick composites, the electrical conductivity after being heat pressed is approximately 10^5^ S m^–1^ but drops more significantly at a thickness of 3 mm, which may be a result of the LM droplets having a greater volume in which to settle and coalesce. This coalescence could cause the liquid metal to be expelled from the thicker sample when heat pressed, thus decreasing the electrical conductivity.

Finally, flipping the film during the 24 h solvent evaporation process was done to better disperse the LM droplets throughout the polymer matrix and prevent settling of the droplets to one side of the composite. Statically evaporated samples had very few individual, dispersed droplets as most of the liquid metal aggregated or coalesced into a single continuous volume at the bottom of the mold (Figure S3). During this flipping process the surface initially on the bottom of the mold becomes conductive (the 1 and 2 h flips are exceptions to this), and this conductivity is maintained through the evaporation process and into the final solid film (Figure S4). Figure 2d shows the final conductivity of LM-TPU composites with 1.5 mm thickness which were flipped at different times from 1 to 8 h after initial casting. Composites flipped at 1 h showed notable settling of LM droplets similar to samples that were never flipped, as the solution does not change notably from 0 to 1 h. At 2 h the viscosity of the samples was putty-like, causing the composites to have permanent tears from the flipping process which resulted in nonconductive samples. Waiting 3–7 h before flipping resulted in composites that still exhibited some settling and varying levels of putty like behavior, causing the conductivity of the LM-TPU composite to be lower. Flipping the LM-TPU composites at 8 h produced samples that, after the 24 h solvent evaporation, were conductive on the surface and had a homogeneous dispersion of LM droplets (Figure S2b).

The processing parameters of these LM-TPU composites have a notable effect on their mechanical and electrical properties. Through the processing parameters utilized above, we are able to create LM-TPU composites that are electrically conductive as prepared without postprocess sintering. The electrical conductivity likely occurs during the peeling process when removing the materials off of the fabrication substrates. Through optimal microscopy, we find that both the top surface (nonconductive) and bottom surface (conductive) have a similar appearance (Figure S4), showing that LM is not left on the surface and that the conductive network is embedded within the composite material. Through this embedded LM network, the conductivity is comparable to or higher than other mechanically sintered LM composites^66,67,71,72^ or LM fibers.^20,23,26^ Additionally, the thermal reprocessing which enables the iron-on aspects of our materials facilitates adhesion and integration into fabrics and with rigid electrical components. This combination of electrical conductivity and adhesion enables robust and rapid fabrication of wearable devices into textiles.

### Soft Circuit Integration into Fabrics

In addition to being electrically conductive, these composites also integrate into fabrics. By using thermoplastic polymers such as TPU that can act as hot melt adhesives, LM-TPU composites can be adhered to fabrics of varying weaves via heat transfer.^31^ While other studies have shown LM adhesion to fabrics,^26,73–75^ this thermal transfer approach is unique to thermoplastic elastomer composites due to their reprocessability, enabling films to be made and then robustly integrated into fabrics through heat pressing. The main types of weaves evaluated in this study are plain weave (Polyester), twill (Cotton), knit (Spandex), and a mesh weave (Jersey-Mesh) intended for athletic wear. The LM-TPU composites were heat pressed with fabric on both sides of the composite at 177 °C at a pressure of 16.5 kPa for 15 min. The temperature, time, and pressure were chosen as they allow the LM-TPU composite to melt and flow into the fabric without rupturing the outside of the composite.

Once the conductive fabrics were created, adhesive fracture energy was measured using a T-peel test^76^ (Figure 3a). The fracture energy of the LM-TPU composites on different fabrics is dependent on the type of fabric, its weave, and the volume fraction of the LM within the composite. The weave and material of each fabric provide varying interfaces for adhesion, altering the ability of the composite to both infiltrate and interlock within the fibers due to different weave densities, as well as providing different chemical interactions between the composite and fabric material. Additionally, the surface patterning of the fabric that results from the warp and weft of the weave leads to a fracture energy that exhibits a cyclic pattern as the crack travels across the length of the adherends^77^ (Figure 3b). We see with the cotton fabric that there are large peaks and valleys, especially for LM composites with lower volume fractions. Therefore, fracture energy for these composites was determined as an average of the plateau region of the T-peel tests. The volume fraction of LM is tied to the cohesive strength of the adhesive layer; while the adhesion in this system fails at the interface between fabric and adhesive, this is still affected by the cohesive strength of the composite, as the deep infiltration and mechanical interlocking of the composite between fibers requires the fracture of some composite to debond the adhesive structure (Figure S5).

**Figure 3:**
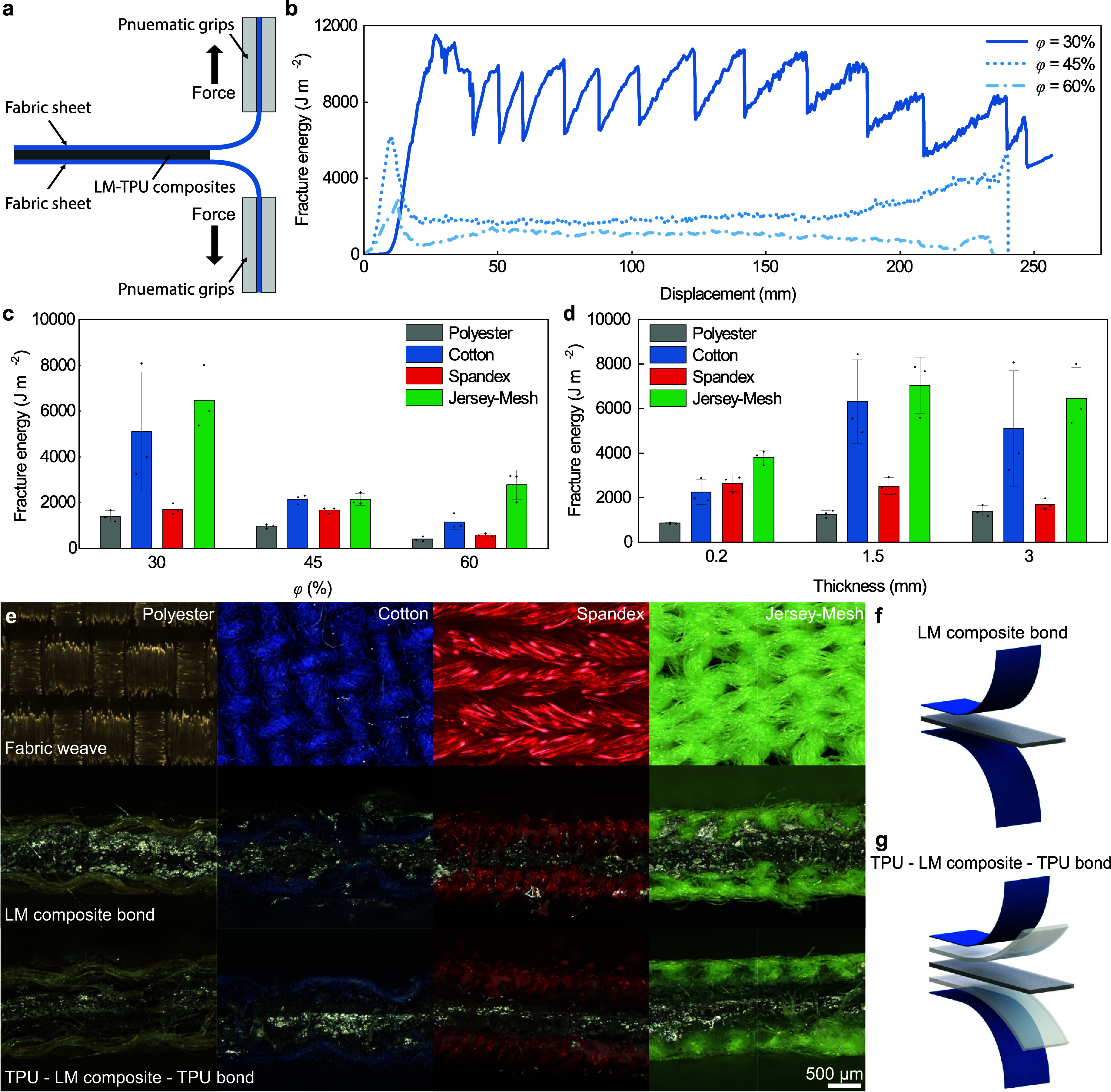
LM-TPU composite adhesion to fabric. (a) Schematic visual of the T-peel testing setup for fabric adhesion using pneumatic Instron grips. (b) Force–displacement curve for cotton fabric and varying LM volume percents. (c) Fracture energy of fabrics by T-peel testing at 3 mm thickness based on varying LM volume fraction. (d) Fracture energy of fabrics by T-peel testing at ϕ = 30% with varying composite thickness. (e) Optical images of T-peel cross sections, varying fabrication methods on different fabric materials and weaves. (f) Schematic render of the fabric adhesion layup for LM-only adhesive structures. (g) Schematic render of the fabric adhesion layup for TPU-LM composite-TPU adhesive structures. Data represent the mean ± 1 s.d. (*n* = 3).

Fracture energy was measured based on two parameters: the first was volume fraction of LM (Figure 3c), and second was the thickness of the adhesive layer (Figure 3d). The volume fractions tested were ϕ = 30, 45, and 60% across the 4 fabrics of interest at a thickness of 3 mm. To measure how the adhesion changed based on thickness, ϕ = 30% volume fraction composites were investigated at thicknesses of 3, 1.5, and 0.2 mm. As a general trend, the ϕ = 30% composites tended to have the highest measured fracture energy, and fracture energy decreased as the volume fraction of LM increased, as previously observed in LM composites.^78^ The ϕ = 60% composite still provided significant adhesion (2720 ± 520 J m^–2^ for Jersey-Mesh) to fabric, making it useful for adhesive circuits. We see in both graphs that thinner fabrics with more fibers had higher adhesion toughness as more composite is able to adhere to individual fibers and create a fully infiltrated surface. The cotton and jersey-mesh samples had a fabric thickness of 0.45 mm and the Spandex had a thickness of 0.58 mm. The polyester fabric provided low adhesion, which is likely due to the water resistant polyurethane coating already applied to the fabric.

The thickness of the adhesive layer also has an effect on adhesive fracture energy. Increasing thickness from 0.2 to 1.5 mm showed a notable increase in fracture energy from 3750 ± 310 J m^–2^ at 0.2 mm to 6930 ± 1240 J m^–2^ at 1.5 mm for Jersey-Mesh), though a further increase to 3.0 mm did not increase fracture energy further (6350 ± 1350 J m^–2^). This suggests that there is a limit for improving adhesion through increases in thickness, likely due to the amount of composite infiltration possible between fibers of the fabric, the known thickness effects on fracture energy,^79^ and the fact that these fail at the fabric-composite interface. This maximum infiltration depth also provides an explanation for why the Spandex has a higher adhesion than the cotton for 0.2 mm thick samples, where there is not enough TPU composite to fully infiltrate any of the fabrics. Similarly, allowing for full infiltration of the fabric with the 1.5 mm samples increases the fracture energy providing greater or equal fracture energies to similar studies using EGaIn and SIS.^68,69^

When adhering LM-TPU composites, two bonding scenarios were evaluated (imaged in Figure 3e). The first approach, used for the material characterization above, is bonding LM-TPU directly to fabric (Figure 3f) to create e-textiles. Bonding the LM-TPU composite directly to fabric provides good adhesion through infiltration of the composite into the fibers of the fabric. In this method, the LM composite fully infiltrates the entire thickness of the fabric. This infiltration created conductivity throughout the fabric volume and on the surfaces, including on the opposite side from where the composite was originally placed, as seen in the second row of Figure 3e. In the second approach, an encapsulation layer is added which prevents the LM-TPU composite from infiltrating the entire thickness of the fabric, where instead, the pristine TPU flows into the fabric and the LM-TPU bonds to the pristine TPU (Figure 3g). This layout may be more appropriate for wearable devices. To achieve this, a layer (≈ 200 μm) of pristine TPU was added between the fabric and the LM-TPU composite on both sides (Figure 3e, third row) via heat pressing. By placing the pristine TPU polymer on either side of the LM-TPU composite prior to heat pressing, we found that the pristine polymer integrates well with the fabric, creating an encapsulation layer for the LM-TPU composite.

### Electromechanical and Mechanical Properties

Electromechanical properties were evaluated under tension. Normalized resistance (*R*/*R*_0_) versus strain was used to determine how deformation influenced resistance, where *R* is the resistance at a specific strain divided by the composite’s initial resistance (*R*_0_) at 0% strain (Figure 4a). Here, the composite was tested under five conditions: an as-prepared composite without heat pressing, a heat pressed composite without fabric, a heat pressed composite with fabric on one side, a heat pressed composite with fabric on both sides, and a fully encapsulated circuit. For all five conditions the change in *R*/*R*_0_ is directly related to the sample’s initial *R*_0_ prior to straining, where higher *R*_0_ tended to show decreasing or constant resistance during stretching and lower *R*_0_ showed increasing resistance. Taking the initial *R*_0_ of each sample and comparing it to the *R*/*R*_0_ at break shows that the final resistance of all five conditions tends to be around 0.95 ± 0.40 Ω. We attribute this behavior to the connectivity of the LM droplets before and during deformation. For samples with higher *R*_0_, fewer droplet connections are present, and then during deformation new droplet connections are made. This can lower or maintain the resistance during stretching, despite predictions from Pouillet’s law showing a decrease if resistivity is constant.^57^ This is consistent with prior work that showed that applying strain increases LM droplet connections,^56^ and thus a constant or decreasing resistance during deformation.

**Figure 4:**
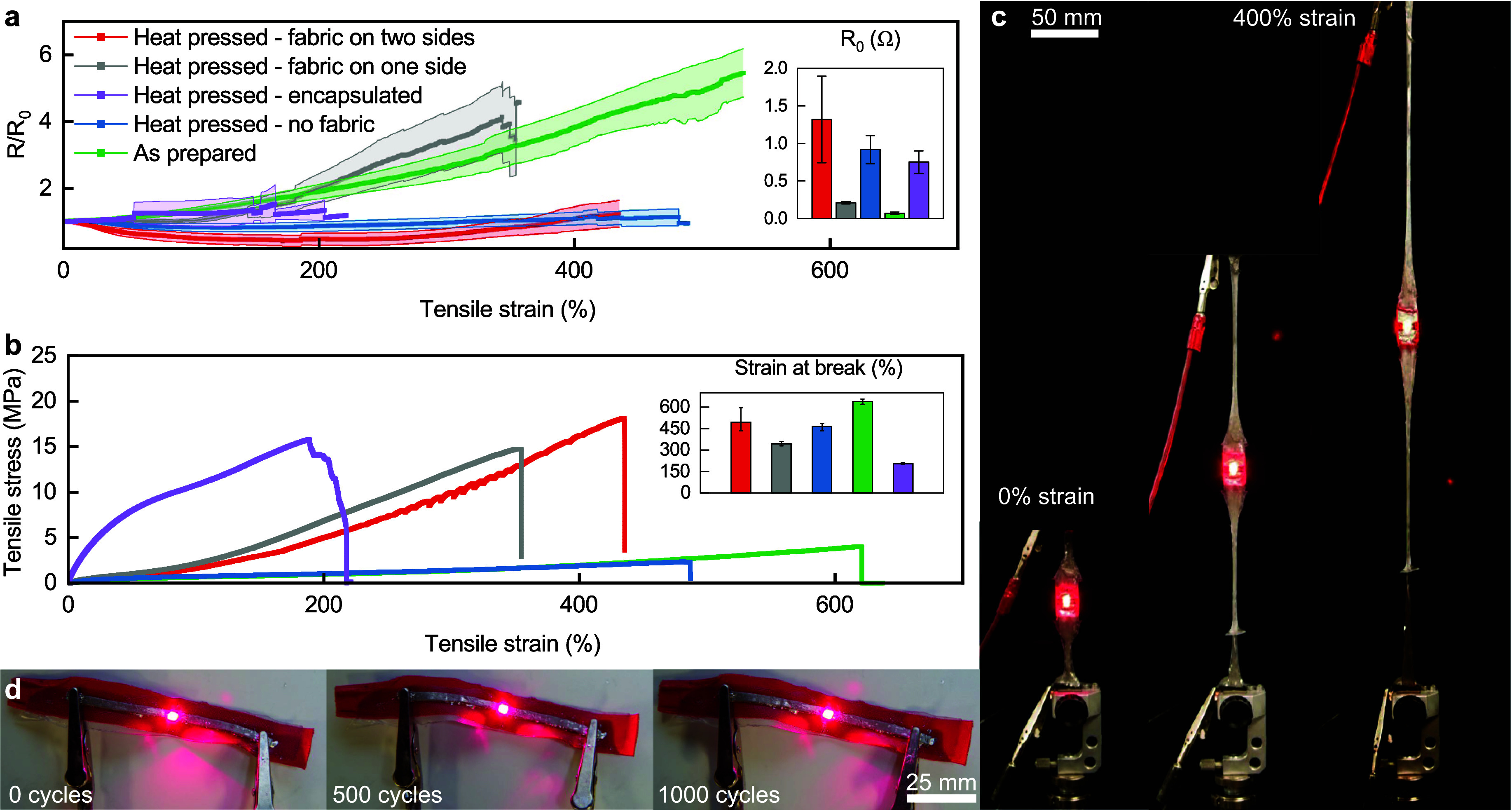
Electromechanical properties of LM-TPU composites. (a) Normalized resistance versus strain for LM-TPU composites under five conditions, where data represent the mean and the shaded areas are ± 1 s.d. (*n* = 3). (b) Stress versus strain curves with strain at break for LM-TPU composites under five conditions. (c) Encapsulated LED circuit showing conductivity at high strain. (d) Images of a LED circuit after the cyclic testing of a LM-TPU composite circuit. Data represent the mean ± 1 s.d. (*n* = 3) in figure insets.

Similarly, stress versus strain measurements were used to determine the mechanical properties of the composite and how heat pressing affected the composite’s elasticity and strain at break (Figure 4b). Adhering fabric to the composite increased the stress required to strain the composite but also lowered the strain at break as the fabrics are less extensible than the elastomer composite. Even without fabric, heat pressing the composites generally decreased the strain at break. Specimens after heat pressing tended to break between 350 and 500% strain compared to the >600% strain at break of the as prepared specimens (Figure S6). The fully encapsulated samples had similar maximum stress compared to the samples with fabric on two sides, but broke around 200% strain due to their increased thickness, suggesting that encapsulation may beneficial for wearable electronics but less so for e-textiles.

Additionally, the deformation of a composite circuit was evaluated through a surface mounted LED connected to a stretchable circuit made of LM-TPU composite (Figure 4c). The LED was first adhered using solvent bonding to a pristine TPU square with an inextensible fabric backing to ensure only the TPU composites would be strained. A dogbone-shaped LM-TPU composite was then adhered to either side of the LED to create a full circuit and a layer of pristine TPU without fabric was heat pressed to encapsulate the circuit. The fully encapsulated circuit withstood strains up to 400%, maintaining LED functionality throughout, before mechanical failure occurred at the interface between the LED and the composite.

Cyclic loading was also evaluated to examine durability of the LM-TPU composite systems. Specimens prepared to the same conditions as in Figure 4a were subjected to repeated tensile loading to 100% strain and showed only minimal change in resistance over 100 cycles (Figure S7). Additionally, a change in *R*/*R*_0_ during the first cycle shows conductivity hysteresis, which is commonly attributed to additional droplet activation effects,^57^ which can be observed in the plots in Figure S7b,c. Another circuit was fabricated by adhering two LM-TPU composite strips to a piece of Spandex with a pristine TPU barrier between the Spandex and LM composite. Again a LED was solvent bonded to the circuit, but no encapsulation layer was added. This circuit was then cyclically folded 270° (135° in either direction) over 1000 cycles to test the durability of the circuit during cyclic loading (Video S1). After 1000 cycles, the damage to the circuit is negligible, and the LM-TPU composite remained electrically conductive and attached to the fabric (Figure 4d). The electromechanical resiliency of this circuit construction highlights the durability, flexibility, and robustness of these LM-TPU composites as conformable wiring for wearable electronics.

### Integrated Wearable Fabric Circuits

Audio sensing is important for applications ranging from voice recognition for on-demand language translation (Figure 5a) to health monitoring of vital signs and human-machine interaction for assistive devices.^80,81^ One common device used in a range of industries are microphones, which need to be readily available, have low noise sensitivity, and be easy to use. Additionally, since it is common for the user to walk around while speaking, it is also beneficial for microphones to be hands free. Previous work has shown that LM-textile integrated devices are resilient to strain both under bending,^26^ and stretching,^68,73,75^ however, there is little work on how these devices compare to more traditional equipment. Therefore, by utilizing the high conductivity, elasticity, and flexibility of the LM-TPU composites we demonstrate a wearable fabric integrated microphone as an easy access assistive device with comparable functionality to standard devices. To demonstrate the application of these fabric composite circuits, a wearable, fabric-integrated microphone was created. A wired miniature electret microphone is used that can be biased using a 2 V source with a 2.2 kΩ impedance and a 1 μf capacitor to the signal output (Figure 5b). The microphone circuit was made using LM-TPU composite wiring within a pristine TPU negative structure, with designated spaces for the rigid components to be placed. This combination was then heat pressed using a household clothing iron. The microphone itself is adhered to the top of the shirt on the right breast and the wiring goes down the front of the shirt and around to the back where it is connected to an external recording device (Figure 5c).

**Figure 5:**
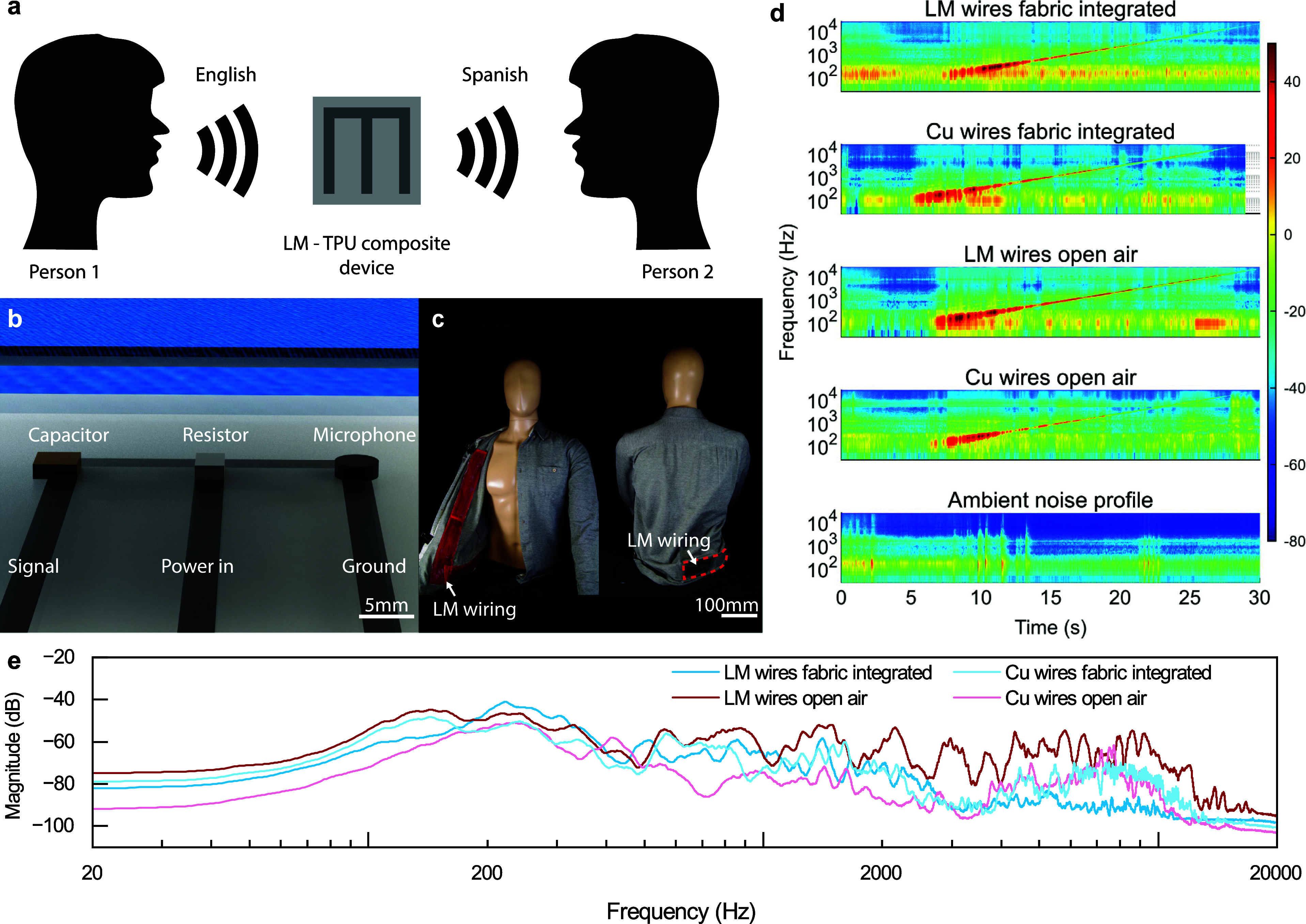
Demonstration of fully encapsulated LM-TPU composite fabric integrated microphone. (a) Schematic of wearable device application. (b) Schematic of microphone circuit with expanded view of rigid components. (c) Images of LM-TPU composite microphone circuit adhered on shirt. (d) Spectrogram of microphone frequency versus time at 2 m with frequency intensity as a color gradient. (e) Intensity of microphone sound at each frequency at a distance of 2 m.

Audio testing was done using Audacity software for four different microphone configurations: the LM wire microphone fully integrated into a fabric shirt worn on a mannequin, a traditional copper wire microphone integrated with fabric, a LM wire microphone in open air, and a copper wire microphone in open air. The fully integrated microphones were surrounded by fabric and TPU, while the open air microphones had no physical barrier between their microphone and the speaker. A speaker (Dell USB Soundbar AC511) was used to run a 20 Hz - 20 kHz (standard human hearing range) frequency sweep across the microphones at distances of *L* = 1, 2, 4, 6, and 8 m in an open warehouse (Figure S8). Figure 5d shows spectrograms of the frequency sweep for each microphone versus time, as well as an additional spectrogram of the noise profile for the room where the recordings took place. The ambient noise profile of the warehouse (AC units, machinery, etc.) was recorded by the open air, copper wire microphone. The fabric integrated, copper wire microphone and the open air, LM wire microphone were used to understand if differences in attenuation occurred due to signal transmission through LM wiring or obstruction of the microphone by fabric. Additionally, the fabric integrated microphone was extended to 50% strain to determine the effect of changing *R*/*R*_0_ on the microphone’s performance. We found that at 0 and 50% strain the LM-TPU composite microphone is able pick up sound equally well, which can be attributed to the composite’s low *R*/*R*_0_ change (Figure S9). All the microphones are able to pick up the full range of frequencies, although intensity of the noise tends to drop at higher frequencies. We also observe ambient noise at low frequencies, especially in the fabric integrated, LM wire microphone; however, this noise does not affect the overall sound quality of the microphone. Video S2 shows the warehouse setup for the microphone at 2 m with the 20 Hz - 20 kHz frequency sweep playing.

Figure 5e shows the intensity of each microphone at each frequency of the sweep at a distance of 2 m between the microphone and speaker. The graphs remain relatively similar until the 2000 Hz range, where the fabric integrated, LM wire microphone sees a decrease in intensity compared to the other microphones due to it being covered in TPU, which can reduce the sound being picked up. It was found that changing the distance had little effect on any of the microphones’ sensitivity and frequency response to signals (Figure S10). Overall, the fabric integrated, LM wire microphone was able to function well compared to traditional microphones even at distances up to 8 m away. The LM-TPU wiring is thus comparable with standard equipment while being more conformal to the user providing added comfort and ease of use.

## Conclusions

A soft LM-TPU composite which is electrically conductive without postprocessing was created using a thermoplastic matrix. These LM-TPU composites enable stretchable conductors that can be readily integrated into a variety of fabrics through heat transfer processes for e-textiles and wearable electronics. This further allows for the incorporation of rigid electrical components which can enhance device functionality. With this process, wearable circuit patches could be heat pressed into clothing without the need for sewing or stitching, enabling on-the-fly, robust integration of wearable devices. Robust electrical and mechanical integration through these LM-TPU composites can be used in fields such as e-textiles, soft circuits, wearable electronics, and soft robotics.

## Methods

### Fabrication of LM TPU Composites

A solution of 35% volume TPU pellets (Covestro RxT 70A) in tetrahydrofuran (THF) was prepared in a mixing cup (∼1:1.5 by weight) and mixed in a dual asymmetric centrifugal mixer (Flacktek DAC 1200–500 VAC SpeedMixer) at 1600 rpm for two 7.5 min cycles, cooling the cup with cold water in between. THF was then added to the container to replace any amount that had evaporated, and the solution was mixed at 1600 rpm for another 4 min. EGaIn was then added into the solution for ϕ = 30, 45, or 60%, hand mixed to disperse evenly, then mixed in the Flacktek at 1600 rpm for 2.5 min.

### Adhering Composites to Fabrics

The LM composites were placed between two sheets of fabric and then placed between two Teflon plates before being put into a Carver Bench Top Model 4120 Heated Press with both platens set to 177 °C. The composites were pressed at 16.5 kPa for 15 min. The composites were removed from the press and allowed to cool before trimming off any excess LM-TPU composite with a hand-held rotary blade. The LM-TPU composite-TPU bonds were made using the same process with the addition of two sections of pristine TPU placed between the LM composite and the two sections of fabric on either side.

### Electrical Conductivity Measurements

All conductivity tests were done using a Keithley 2461 SourceMeter using a four-point Ohmmeter test to determine the resistance through the specimen at *l* = 10, 20, and 30 mm spacings (Figure S1b). Once the resistances had been measured, the conductivity was determined through the following equation using the length of the specimen (*l*), its measured resistance (*R*), and its cross-sectional area (*A*): $\sigma =\frac{l\left(m\right)}{R\left(\Omega \right)A\left(m^{2}\right)}$ .

### T-Peel Testing Procedures

T-Peel tests were performed based on an Instron 5944 universal testing machine with universal tensile grips and a 2 kN load cell. T-peel samples were prepared through heat pressing, then cut to 3 specimens 25 mm wide by 175 mm long, bonded over 100 mm of their length. Adhesive fracture energy (*G*_c_) was calculated using $G_{c}=\frac{2F}{w}$ , in which *F* is the measured load (averaged critical force) and *w* is the width of the specimen (25 mm).

### Electromechanical Testing

Electromechanical testing was performed in an Instron 5944 universal testing machine. LM-TPU samples were die cut into dog bone specimens at 50% of ATSM D412 type C standard size. A strip of copper tape was placed on either end of the dog bone specimens which connects to a Keithley 2461 SourceMeter measuring electrical resistance. Specimens were tested at a rate of 60 mm/min until fracture.

### Wearable Microphone Fabrication

First, four pristine TPU films were cast at 200 μm thickness, two pristine TPU films at 3 mm thickness, and one LM-TPU composite film at 3 mm thickness into molds 50 mm wide by 560 mm, leaving them to evaporate solvent for 24 h. Two of the strips of 200 μm thick pristine TPU were pressed into strips of the Spandex the same length and width as the TPU, while the remaining strips of 200 μm thick pristine TPU were pressed into an L-shape on the inside of a button-down shirt. Two strips of 3 mm thick pristine TPU were laser cut into the desired circuit pattern mold, and then cut the LM-TPU into 2 mm wide strips of appropriate length using a rotary blade and cutting mat. Next, the LM-TPU composite and rigid components were placed into the pristine TPU mold, coated with THF using a transfer pipet to bond the TPU, and left to evaporate solvent for 1 h. Finally, the electrical circuit patch was heat pressed to the shirt using a household clothing iron set to 177 °C by consistently sweeping across the length of the circuit to ensure even adhesion. The circuit was covered on the back with the Spandex strips using the household clothes iron.

### Wearable Microphone Testing and Data Processing

The wearable microphone shirt was placed on a mannequin and was tested in an open warehouse environment. During recording the microphone remained stationary while a speaker (Dell USB Soundbar AC511) was used to run a frequency sweep of 20 Hz–20 kHz at distances of 1, 2, 4, 6, and 8 m from the microphone. Sound acquisition for these microphones was recorded and processed using Audacity software. The microphone recordings for the wearable microphone were amplified by 20 dB for every distance.

## Supplementary Material







## Data Availability

The data that support the findings of this study are available from the corresponding author upon reasonable request.

## Notes

General conductivity data for LM-TPU composites; SEM droplet analysis for 1600 and 1000 rpm samples; SEM images of settled droplets within samples flipped at varying times during evaporation; surface images of LM-TPU composites; images of T-peeled samples showing interfacial failure; raw stress–strain data for composite fabric layups; raw data for cyclic electromechanical tests; warehouse setup for microphone demo audio capture; audio data for LM fabric integrated microphone at varying strains; and raw audio data for all microphones (PDF)
LM-TPU composite cyclic bending (MP4)
20 Hz–20 kHz frequency sweep for a LM-TPU fabric incorporated microphone at 2 m (MP4)
