# Supplementary material for: Iron-On Wearable Electronics through Liquid Metal Adhesive Composites
Source: ACS Appl Mater Interfaces. 2025 Nov 9;17(46):63764–74. doi: 10.1021/acsami.5c13752 (PMC12635972; doi:10.1021/acsami.5c13752)
Supplement: Supplementary file 1 [file am5c13752_si_001.pdf]

Supporting Information

Iron-On Wearable Electronics through Liquid Metal Adhesive Composites

John Joyce[1], Brittan T. Wilcox[1], Anna Ingram[1], and Michael D. Bartlett[1,2*]

[1]Mechanical Engineering, Soft Materials and Structures Lab, Virginia Tech, Blacksburg, VA 24061, USA.

[2]Macromolecules Innovation Institute, Virginia Tech, Blacksburg, VA 24061, USA.

[*]Corresponding author email: mbartlett@vt.edu

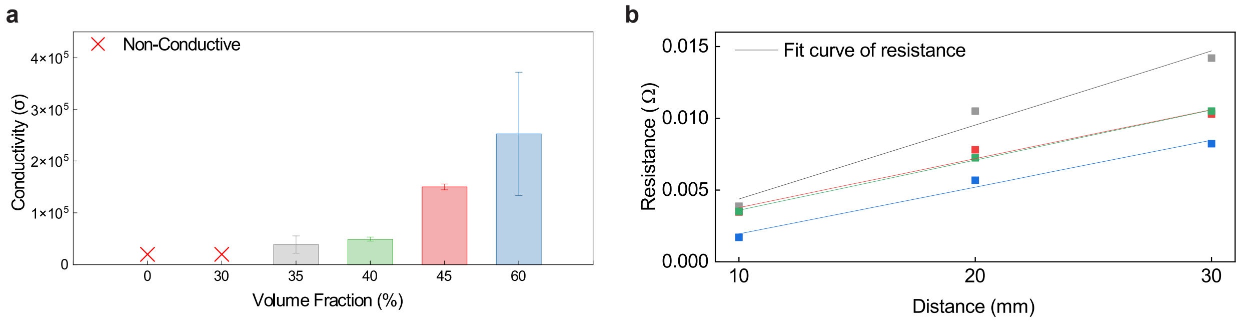

Fig S1 Electrical conductivity of LM-TPU composites. a) Conductivity of of as-prepared composites for 3mm thick LM at varying volume percents. b) Raw resistance data for $\phi = 45\%$ LM-TPU composite.

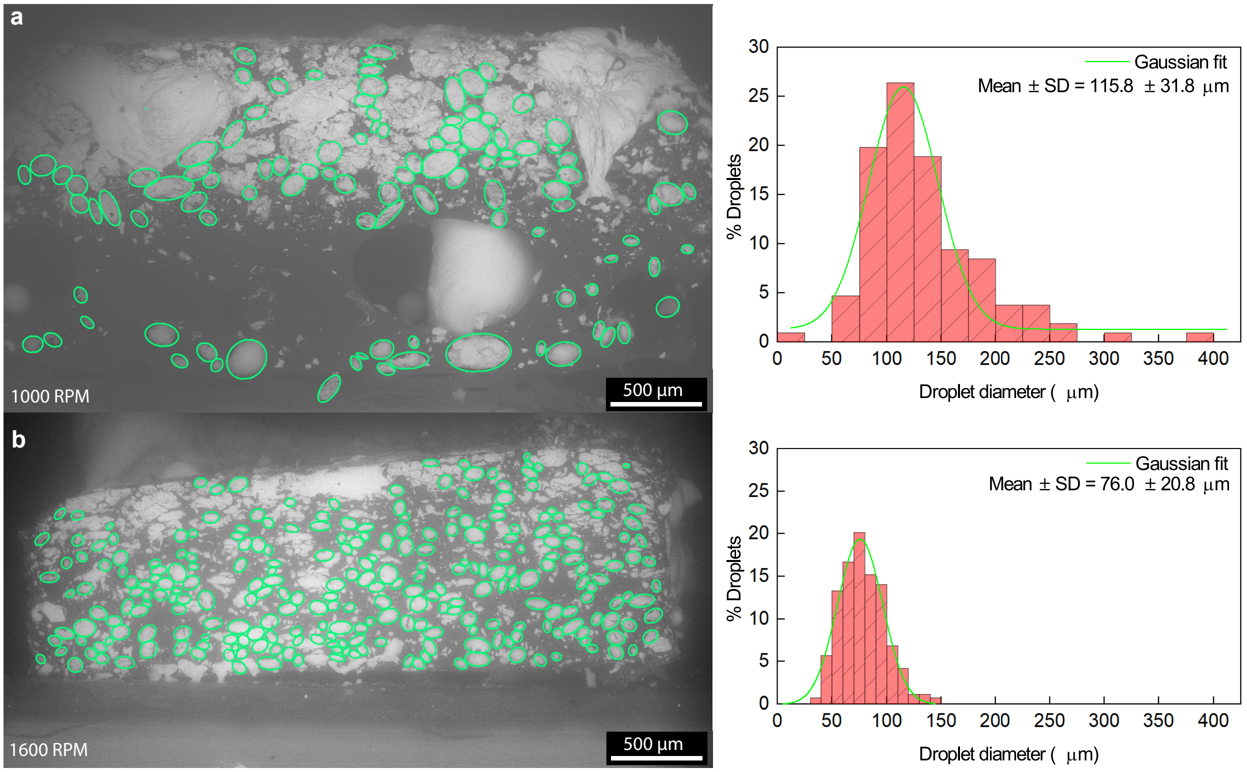

Fig S2 Particle analysis of LM-TPU composites. a) SEM image of composite cross section mixed at 1000 RPM with histogram of droplet sizes. b) SEM image of composite cross section mixed at 1600 RPM with histogram of droplet sizes.

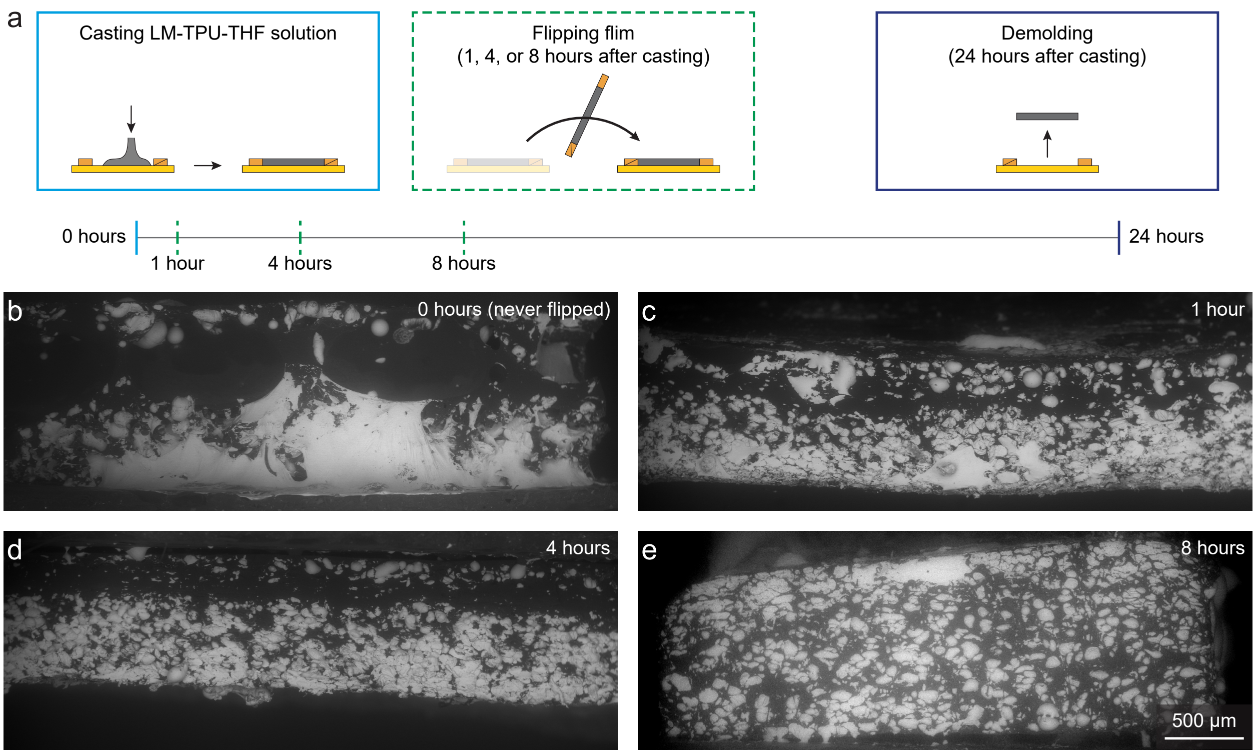

Fig S3 (a) Schematic of flipping during fabrication. SEM images of LM-TPU composites that are (b) not flipped during solvent evaporation, and those flipped at (c) 1 hour, (d) 4 hours, and (e) 8 hours.

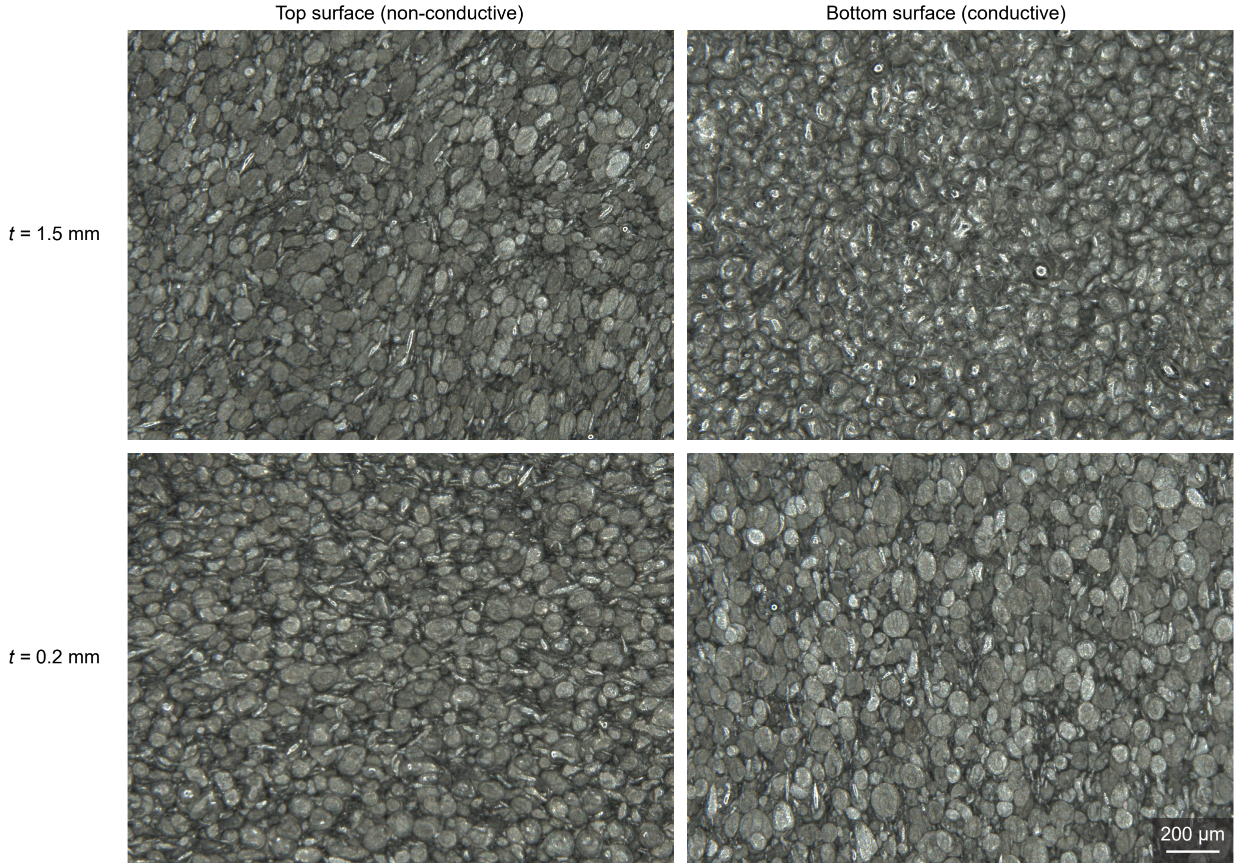

Fig S4 Optical microscopy of film surfaces for $\phi = 60\%$ LM-TPU composites, showing both the conductive and non-conductive surfaces for both 1.5 mm and 0.2 mm thick films.

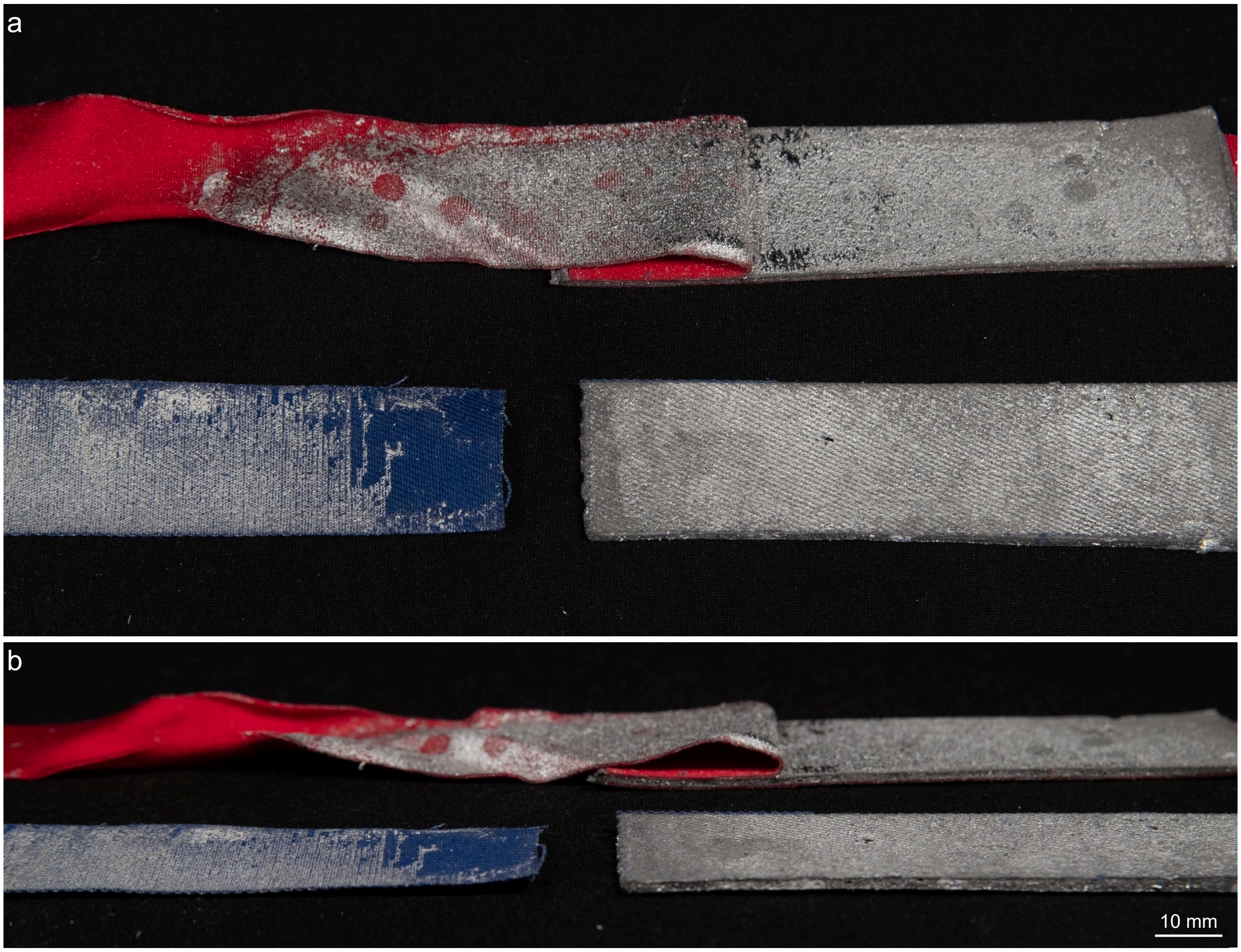

Fig S5 Images of T-peel specimens after testing showing interfacial failure mode, from a) above and b) the side.

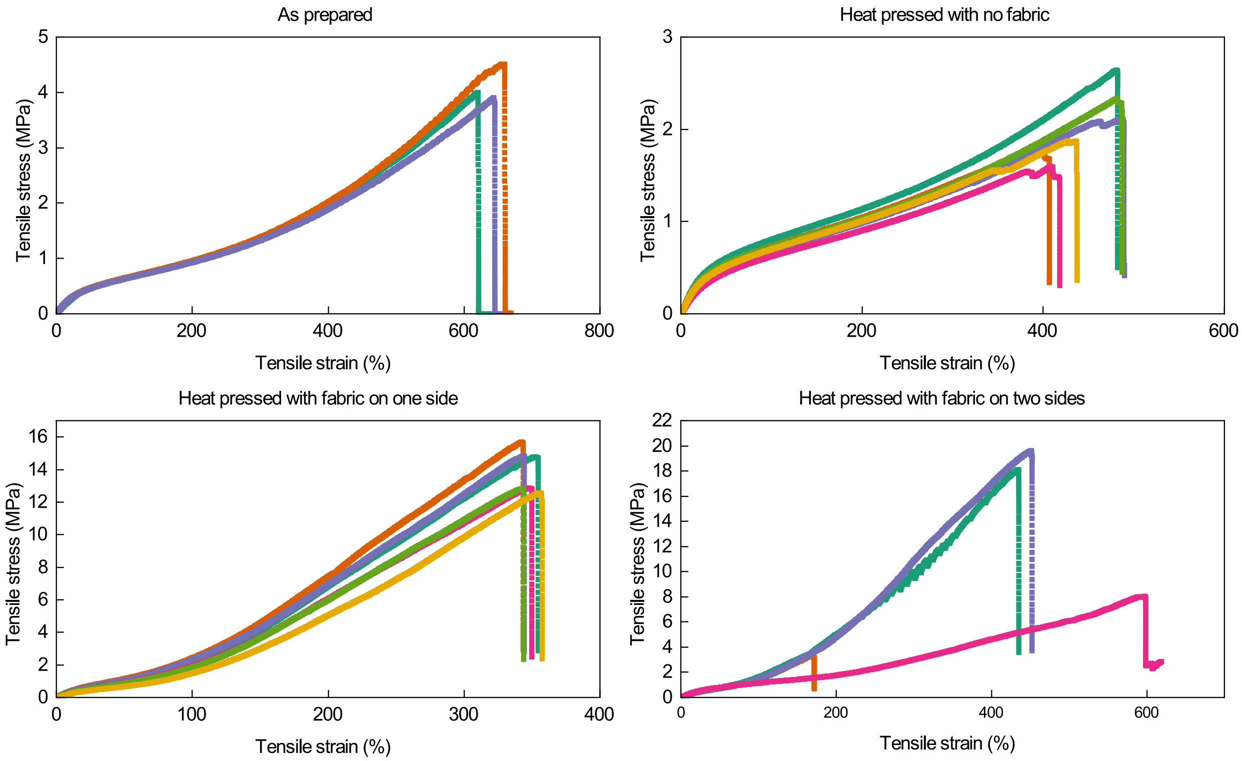

Fig S6 Stress-strain data for composite-fabric layup used in electromechanical characterization.

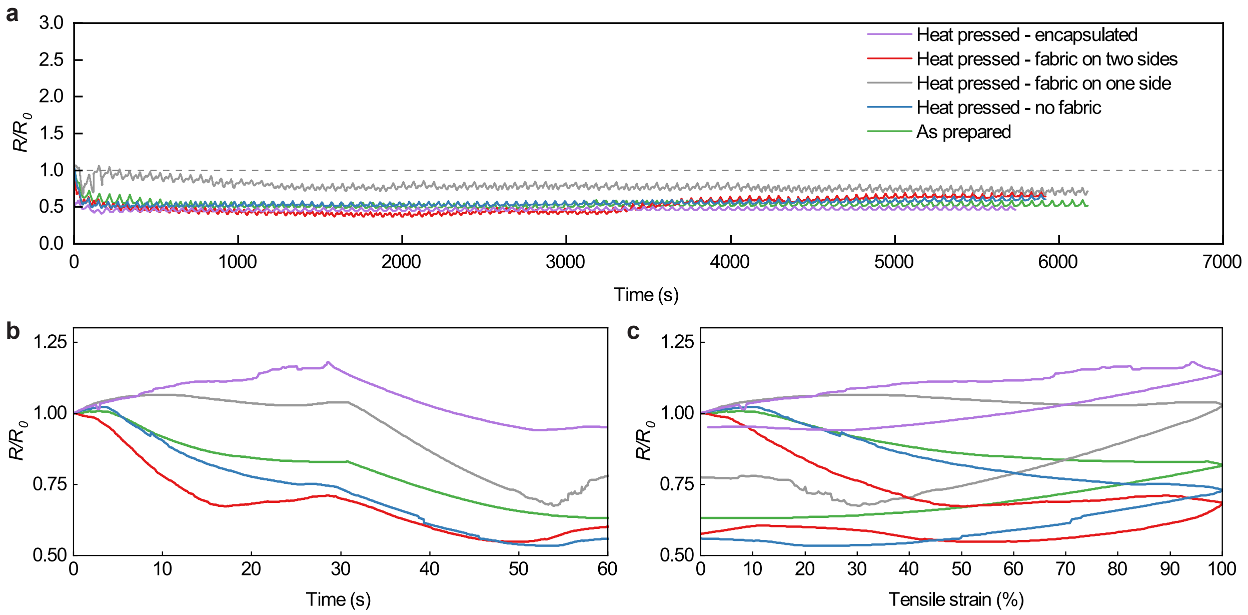

Fig S7 a) Normalized resistance for LM-TPU composites under five conditions exposed to 100% strain for 100 cycles, b) Normalized resistance over time for the first cycle only, c) Hysteresis of normalized resistance over strain during the first cycle.

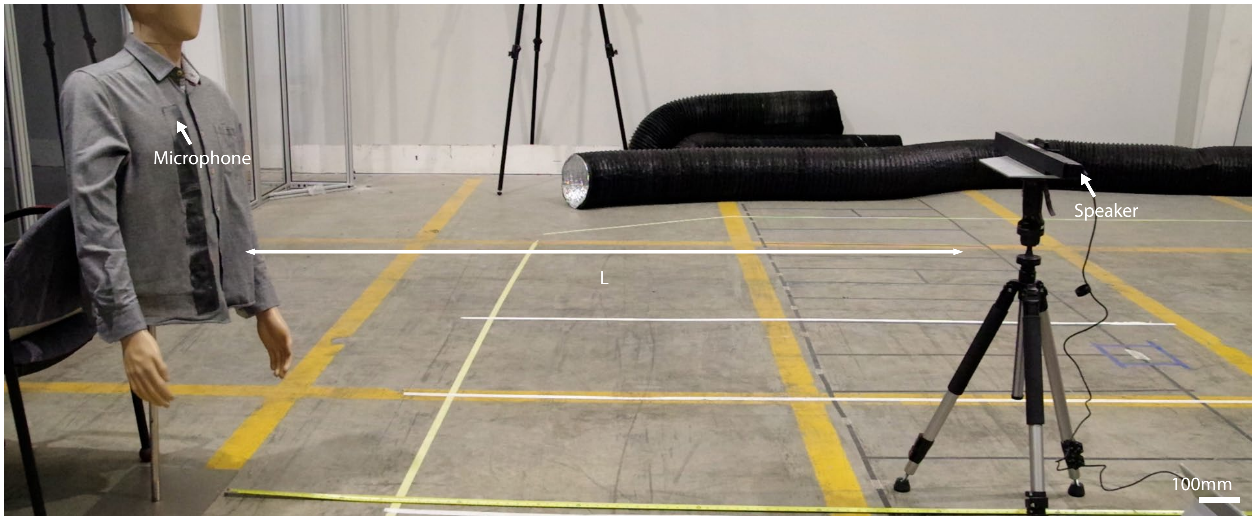

Fig S8 Microphone testing setup in warehouse.

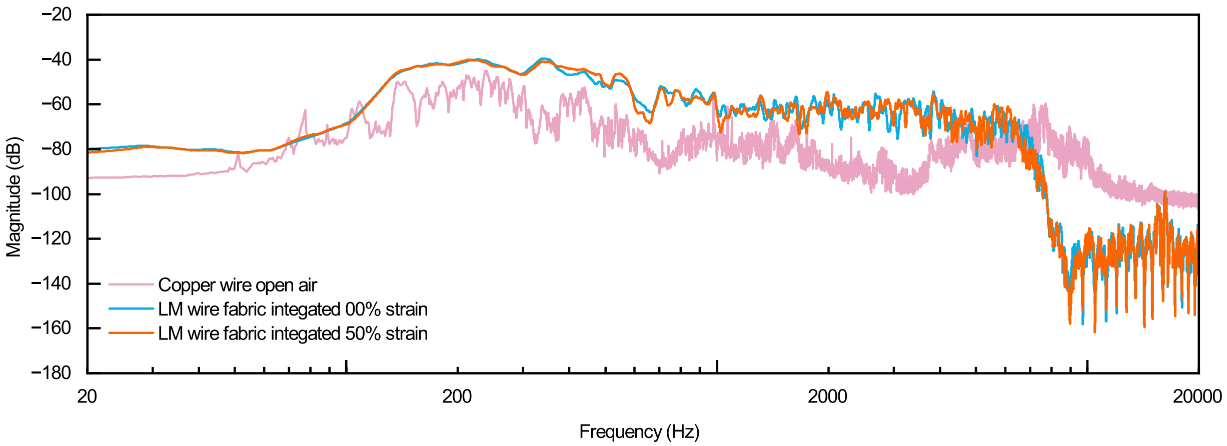

Fig S9 Raw audio data for LM wire, fabric integrated microphone extended to 50% strain.

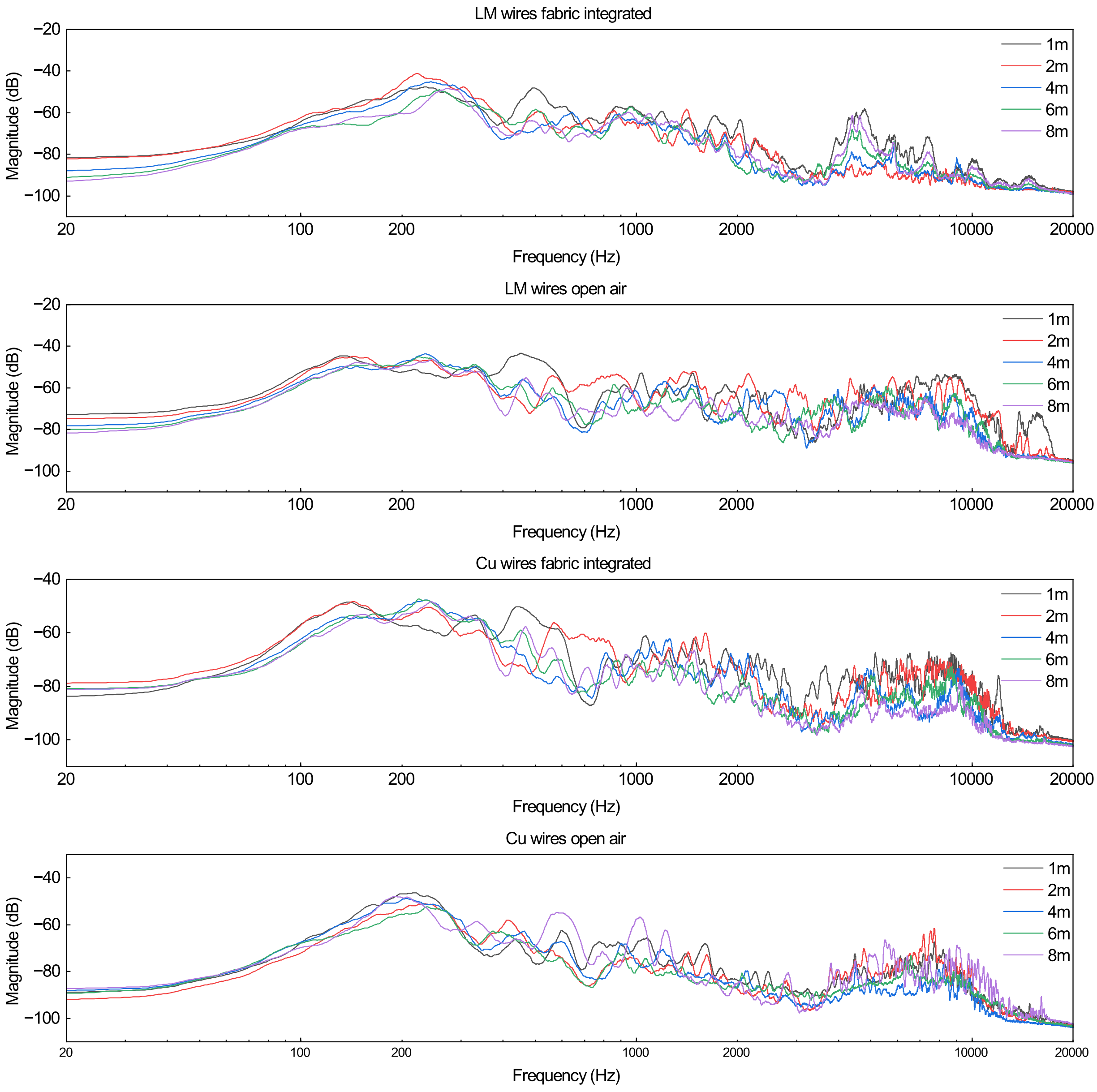

Fig S10 Raw audio data for each microphone circuit at all distances measured.
